# Antibacterial Effects of Chitosan/Cationic Peptide Nanoparticles

**DOI:** 10.3390/nano8020088

**Published:** 2018-02-05

**Authors:** Frans Ricardo Tamara, Chi Lin, Fwu-Long Mi, Yi-Cheng Ho

**Affiliations:** 1 Department of Bioagricultural Science, National Chiayi University, Chiayi 60004, Taiwan; ansardo@gmail.com (F.R.T.); mervynlin11@gmail.com (C.L.); 2 Graduate Institute of Medical Sciences, College of Medicine, Taipei Medical University, Taipei 11031, Taiwan; 3 Department of Biochemistry and Molecular Cell Biology, School of Medicine, Taipei Medical University, Taipei 11031, Taiwan; 4 Graduate Institute of Nanomedicine and Medical Engineering, College of Biomedical Engineering, Taipei Medical University, Taipei 11031, Taiwan

**Keywords:** antimicrobial activity, chitosan, nanoparticle, protamine, biofilm

## Abstract

This study attempted to develop chitosan-based nanoparticles with increased stability and antibacterial activity. The chitosan/protamine hybrid nanoparticles were formed based on an ionic gelation method by mixing chitosan with protamine and subsequently cross-linking the mixtures with sodium tripolyphosphate (TPP). The effects of protamine on the chemical structures, physical properties, and antibacterial activities of the hybrid nanoparticles were investigated. The antibacterial experiments demonstrated that the addition of protamine (125 µg/mL) in the hybrid nanoparticles (500 µg/mL chitosan and 166.67 µg/mL TPP) improved the antimicrobial specificity with the minimum inhibitory concentration (MIC) value of 31.25 µg/mL towards *Escherichia coli* (*E. coli*), while the MIC value was higher than 250 µg/mL towards *Bacillus cereus*. The chitosan/protamine hybrid nanoparticles induced the formation of biofilm-like structure in *B. cereus* and non-motile-like structure in *E. coli*. The detection of bacterial cell ruptures showed that the inclusion of protamine in the hybrid nanoparticles caused different membrane permeability compared to chitosan nanoparticles and chitosan alone. The chitosan/protamine nanoparticles also exhibited lower binding affinity towards *B. cereus* than *E. coli*. The results suggested that the hybridization of chitosan with protamine improved the antibacterial activity of chitosan nanoparticles towards pathogenic *E. coli*, but the inhibitory effect against probiotic *B. cereus* was significantly reduced.

## 1. Introduction

Antibiotic resistance caused by the overuse of antibiotics has become an important and growing problem all over the world. According to the World Health Organization (WHO) and the Centers for Disease Control and Prevention (CDC), the rise of superbugs such as methicillin-resistant *Staphylococcus aureus* (*S. aureus*) (MRSA), multiple-drug resistant (MDR) *Enterobacteriaceae*, *Acinetobacter*, and *Pseudomonas*, and extreme drug-resistant *Mycobacterium tuberculosis* were the most common infectious causes of death over the last few decades [1]. The current strategies used for *s*olving the problem of the growing crisis of antibiotic resistance are mainly centered on the reduction of antibiotic consumption and the development of new antibiotic drugs.

Chitosan is a natural polysaccharide consisting of glucosamine and *N*-acetyl glucosamine units. The cationic characteristic of chitosan allows it to exhibit superior inhibitory activity against a wide variety of microorganisms, including fungi, trypanosomes, and bacteria [2,3,4,5,6]. Positively-charged chitosan molecules might interact with negatively-charged microbial cell membranes, leading to alterations in cell wall permeability and the leakage of intracellular compounds. However, factors including molecular weight, deacetylation degree, and positive charge content can affect the antibacterial activities of chitosan [7]. Some studies have modified chitosan with sulfonate groups or quaternary ammonium groups, and integrated antibacterial herbs or enzymes into chitosan-based beads or nanoparticles to improve their antimicrobial activities [8,9,10].

Protamine is a natural cationic antimicrobial peptide (CAP) composed mainly of strongly basic arginine residues. Protamine has broad-spectrum antimicrobial activities against a wide range of gram-positive and gram-negative bacteria [11,12,13,14]. The antimicrobial mechanism of action for protamine is believed to be the electrostatic attraction between the cationic peptide and the negatively-charged cell envelope, which kills susceptible bacteria due to cell envelope lysis and leakage of K^+^, adenosine triphosphate (ATP), and intracellular enzymes [15,16]. Protamine sulfate was also investigated for use in anti-infective coatings to control biofilm growth on medical devices [14,17,18]. 

Chitosan in combination with protamine has been developed to deliver insulin, DNA, siRNA, and heparin for intensive insulin therapy and gene therapy [19,20,21]. However, chitosan/protamine-based antibacterial nanomaterials have not yet been reported. In this study, *Bacillus cereus* and *Escherichia coli* (*E. coli*) were selected for the antibacterial study because they are frequently used as representatives of gram-positive and gram-negative bacteria, respectively. Furthermore, some strains of *Bacillus cereus* can be beneficial as probiotics for animals. This research aimed to develop a new type of chitosan-based nanoparticle through the combination of chitosan with the cationic protamine, having a higher antimicrobial activity against pathogenic bacteria (such as *E. coli*) compared to the nanoparticles prepared from chitosan alone. The hybrid nanoparticles had a higher antimicrobial activity against *E. coli*, but lower antibacterial activity against *B. cereus*, and are environmentally-friendly and stable over a wide pH range. 

## 2. Results and Discussion

### 2.1. Characterzation of Chitosan and Protamine

**Figure 1:**
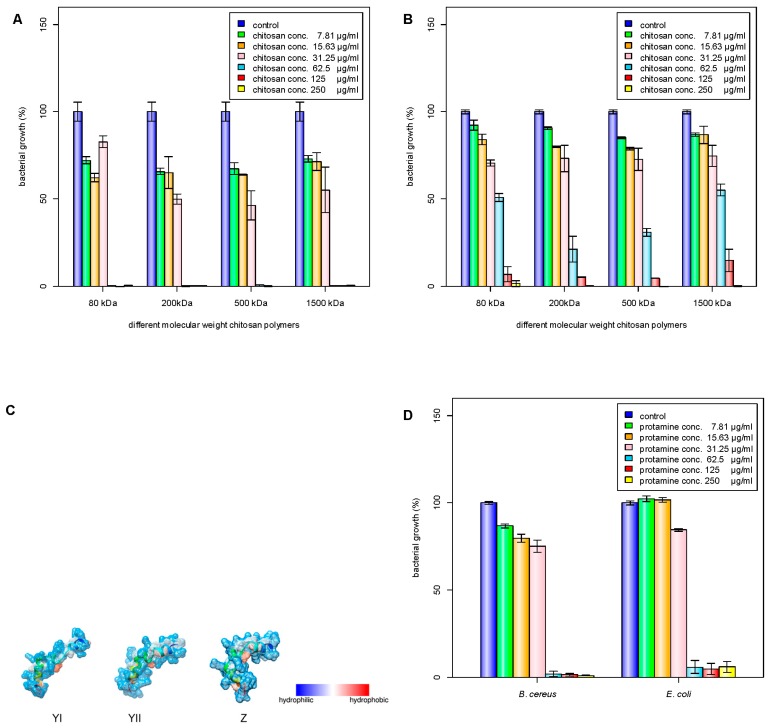
Inhibition of bacterial growth by different molecular weight chitosan polymers. M1 (80 kDa), M2 (200 kDa), M3 (500 kDa), M4 (1500 kDa); C1 (250 μg/mL), C2 (125 μg/mL), C3 (62.5 μg/mL), C4 (31.25 μg/mL), C5 (15.63 μg/mL), C6 (7.81 μg/mL): (**A**) *B. cereus* growth; (**B**) *E. coli*; (**C**) depiction of major protamine components YI, YII, and Z; (**D**) inhibition of bacterial growth by protamine. C1 (250 μg/mL), C2 (125 μg/mL), C3 (62.5 μg/mL), C4 (31.25 μg/mL), C5 (15.63 μg/mL), C6 (7.81 μg/mL). C1–C6 means different concentrations of chitosan solutions.

Chitosan has been investigated for its antimicrobial properties against a wide range of microorganisms. The antimicrobial activity of chitosan is affected by its molecular weight and degree of acetylation independently [3,22], and the molecular weight has a stronger effect on the antimicrobial activity compared to the degree of acetylation [23,24,25]. In acid, the antimicrobial activity was shown to increase with increasing molecular weight [24], while the antimicrobial activity changed at pH 7.0 [7]. In this study, four different molecular weights of chitosan (80, 200, 500, and 1500 kDa) were tested for their antibacterial activities (Table 1). The 200-kDa chitosan was found to have the lowest minimum inhibitory concentration (MIC) against *E. coli* (Figure 1A). On the other hand, *B. cereus* was found to have similar MIC values for all the tested chitosan molecular weights (Figure 1B). It has been reported that decreasing the molecular weight of chitosan may increase its binding affinity to the membrane due to improved mobility, attraction, and ionic interaction [26], though a proper antibacterial activity can be obtained only when the molecular weight is larger than 10 kDa. Generally, protamine consists of 20 arginine molecules from a total of 30 amino acids. The molecular weight of the protamine was about 4 kDa and had a high isoelectric point (IEP) of around 13.3, and low grand average of hydropathicity (GRAVY) value of −2.8. The GRAVY value was calculated by adding the hydropathy value for each residue and dividing by the length of the sequence. A negative value showed that the peptide was hydrophilic. The structures of protamine peptides were alpha helix with hydrophilic surface properties (Figure 1C). Figure 1D shows that protamine at the same concentration was found to have higher antibacterial activity against *E. coli* than *B. cereus*.

### 2.2. Chemical and Physical Properties of Nanoparticles

The 200 kDa chitosan was selected to prepare chitosan nanoparticles because of its lowest MIC value against *E. coli* and *B. cereus* (Table 1). The chitosan nanoparticles were prepared from different concentrations of chitosan, NP1 (250 μg/mL), NP2 (500 μg/mL), and NP3 (750 μg/mL) at a chitosan to sodium tripolyphosphate (TPP) weight ratio of 3:1. As shown in Table 2, the particle sizes of NP1, NP2, and NP3 were 78.4 ± 4.01, 150.67 ± 3.05, and 201 ± 3.60 nm, respectively. The zeta potential values were 33.77 ± 1.30 mV for NP1, 33.63 ± 0.32 mV for NP2, and 32 ± 1.11 mV for NP3 (Table 2). Higher chitosan concentration was shown to positively correlate with the size of nanoparticles; nevertheless, the zeta potential value differences were not readily apparent between NP1, NP2, and NP3. 

Table 2 also shows the mean particle size and zeta potential of chitosan/protamine nanoparticles. According to Table 1, MIC and minimum bactericidal concentration (MBC) values for all the tested chitosan molecular weights were smaller than 250 µg/mL. Thus, we kept the chitosan concentration at 250 µg/mL in all chitosan/protamine nanoparticle formulations. The hybrid nanoparticles were produced by mixing 500 μg/mL chitosan with three different protamine concentrations and a chitosan to TPP ratio of 3 to 1. The addition of 125, 250, and 500 μg/mL in the chitosan/TPP mixture (500 μg/mL chitosan, chitosan to TPP weight ratio of 3:1) produced nanoparticles (NPr1, NPr2, and NPr3) with sizes 114.33 ± 4.16 nm (NPr1), 84.8 ± 2.07 nm (NPr2), and 79.4 ± 1.90 nm (NPr3) (Table 2), and zeta potentials 32.23 ± 0.76 mV (NPr1), 30.27 ± 0.72 mV (NPr2), and NPr3 27.67 ± 1.45 mV (NPr3) (Table 2), respectively. The increase of protamine concentration resulted in the decrease of both diameter and zeta potential of the nanoparticles. The incorporation of more cationic protamine might enable the nanoparticles to be more completely cross-linked with the negatively-charged TPP, leading to the formation of stronger compact complexes by decreasing particle sizes. However, the higher density of the incorporated anionic TPP caused the decrease of the nanoparticle zeta potential.

### 2.3. Antibacterial Effects of Chitosan and Chitosan/Protamine Nanoparticles

**Figure 2:**
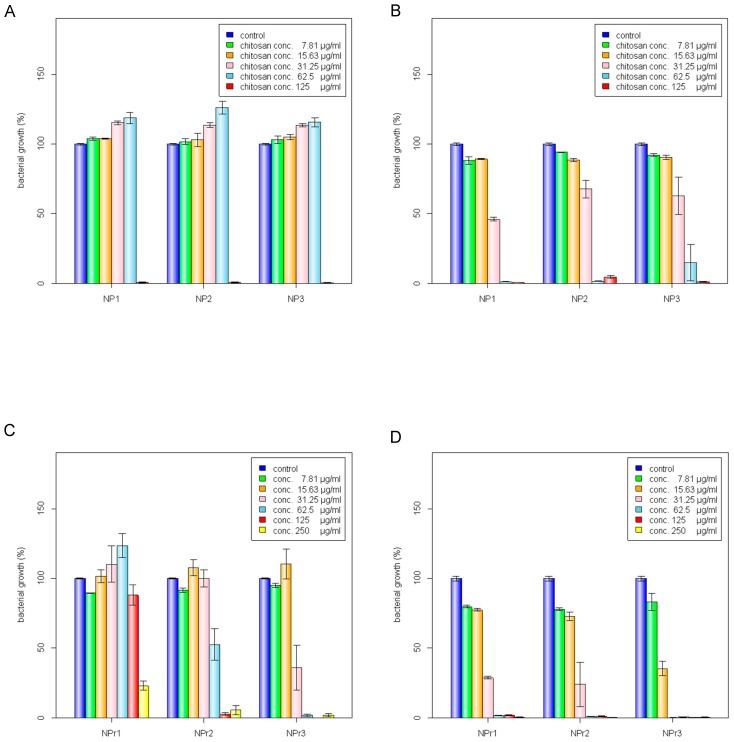

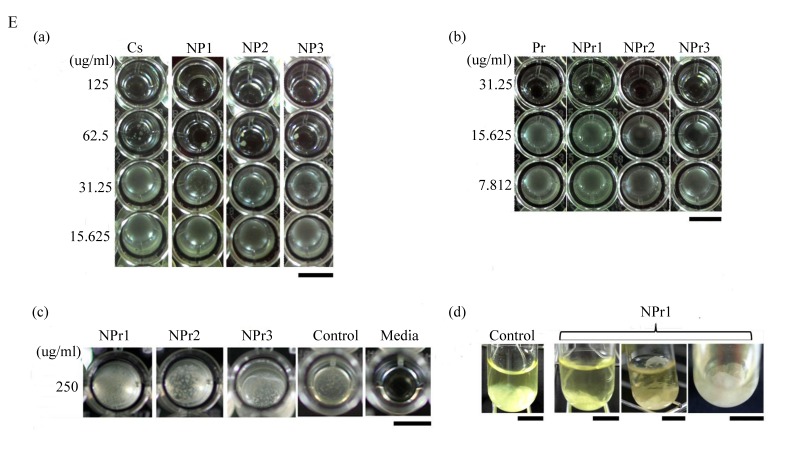
Inhibition of bacterial growth by chitosan nanoparticles (NP) against (**A**) *B. cereus* and (**B**) *E. coli*: C1 (125 μg/mL), C2 (62.5 μg/mL), C3 (31.25 μg/mL), C4 (15.63 μg/mL), C5 (7.81 μg/mL). Inhibition of bacterial growth by chitosan/protamine nanoparticles (NPr) against (**C**) *B. cereus* and (**D**) *E. coli*: C1 (250 μg/mL), C2 (125 μg/mL), C3 (62.5 μg/mL), C4 (31.25 μg/mL), C5 (15.63 μg/mL), C6 (7.81 μg/mL). (**E**) Effect of (a) chitosan nanoparticles treatment on *E. coli* growth; chitosan/protamine nanoparticles treatments on (b) *E. coli* growth, (c) *B. cereus* growth, and (d) biofilm-like formation of *B. cereus*; the scale bar represents 50 mm.

Antimicrobial activities of chitosan polymer solution (CS), protamine (Pr), chitosan nanoparticles (NP), and chitosan/protamine nanoparticles (NPr) were examined by determination of MIC and MBC against gram-positive *B. cereus* and gram-negative *E. coli*. It was shown that *B. cereus* treated with CS alone had the lowest MBC among other antimicrobial treatments (Table 3). MIC or MBC is not truly a single number, but a range depending on the dilution series used during its determination, thus the ranges are broader at higher concentrations. The MIC value is defined as the lowest concentration of a given antibiotic that inhibits the growth of a specific organism, while the MBC value is defined as the lowest concentration that demonstrates a pre-determined reduction (such as 99.9%) in CFU/mL when compared to the MIC dilution. The nanoparticles prepared from 200 kDa chitosan at different concentrations (NP1, NP2, and NP3) were observed to have a similar MIC against *E. coli* and *B. cereus* (Figure 2A,B). However, according to the MIC and MBC values, the addition of protamine increased the antimicrobial activity of chitosan nanoparticles (Figure 2C,D). Chitosan was reported to be positively charged and have higher antimicrobial activity, mainly at pH values below its pKa of 6.5 [7]. Analysis of protamine sequence showed that it has a hydrophilic surface (GRAVY = −2.881) and has pI of 13.3 (Figure 1C). Accordingly, protamine will be positively charged all pHs below its pI value. Therefore, the addition of protamine was expected to increase the hydrophilicity, stability, and effective antimicrobial pH ranges of chitosan nanoparticles. 

Chitosan was found to have similar MIC in the form of polymer solution (CS) or nanoparticles (NP). However, the MBC of chitosan nanoparticles was lower than that in polymeric form (chitosan in soluble state). Soluble chitosan with an extending conformation enables better adsorption onto the bacterial cell surface and then diffusion through the cell wall to cause the disruption of the cytoplasmic membrane [27]. MIC values of antimicrobial treated to *E. coli* were generally lower than those treated to *B. cereus* (Table 3). Adding a lower concentration (125 μg/mL) of protamine to the particle (NPr1) had an opposite effect on the antimicrobial activity of chitosan nanoparticles against *B. cereus* (Figure 2C). At higher concentration (500 μg/mL) of added protamine, the MIC value of NPr3 was lower than that of chitosan in polymeric (CS) and nanoparticles (NP) forms, which shows the increase of bacterial growth inhibition activity. The antimicrobial activity of protamine is associated with its high content of cationic arginine (Arg) residues [13], which can cause cell death due to leakage of K^+^, ATP, and intracellular enzymes [11]. The negative impact of NPr1 on *B. cereus* might be due to some protective factors induced by *B. cereus* which increased the bacterial resistance to the nanoparticles. Treatments of chitosan nanoparticles were found to induce a non-motile-like state in *E. coli (*Figure 2Ea,Eb). When treated with chitosan/protamine nanoparticles, this state appeared in a higher concentration (250 μg/mL) with more apparent early biofilm-like structure compared to the lower concentrations (Figure 2Ec). Treatment of chitosan/protamine nanoparticles led to the formation of well-organized biofilms in *B. cereus* after incubation for 2 days (Figure 2Ed)*,* which might be responsible for the high resistance of *B. cereus*. 

### 2.4. Surface Charge and Hydrophobicity

**Figure 3:**
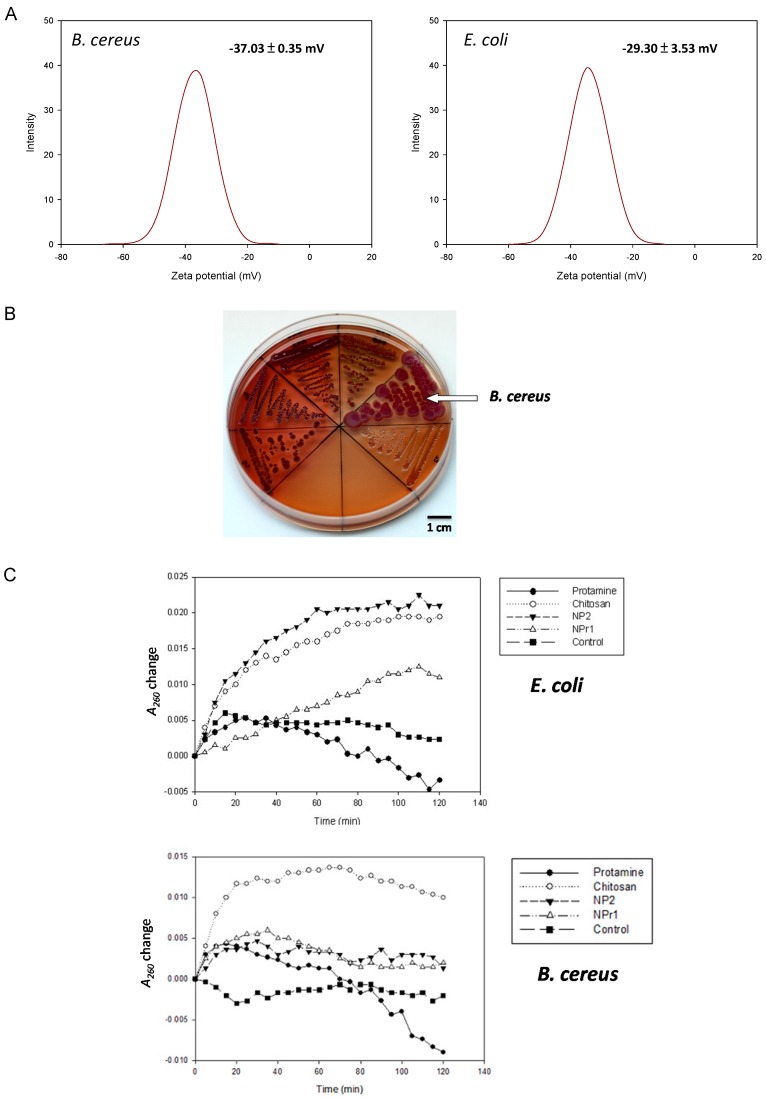

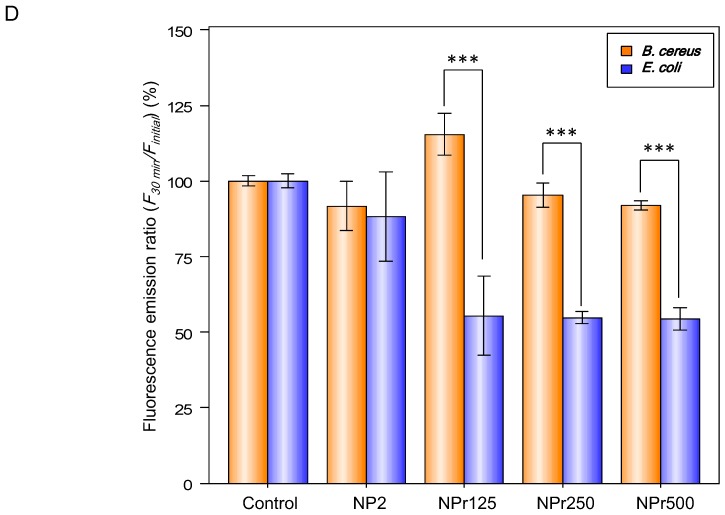
(**A**) Zeta potential distribution of bacterial cells of *B. cereus* and *E. coli*, C4 (31.25 μg/mL), C5 (15.63 μg/mL), C6 (7.81 μg/mL); (**B**) Detection of biofilm production on NB Congo red agar plate (clockwise from the left were *B. subtilis*, *B. pumilus*, *B. amyloliquefaciens*, *B. cereus*, *and E. coli*); (**C**) The release of cell contents identified by absorption at 260 nm from *E. coli* and *B. cereus* by treatment with protamine (125 μg/mL), chitosan (125 μg/mL), NP2 (250 μg/mL), NPr1 (250 μg/mL); (**D**) The binding (30 min)-to-initial (0 min) fluorescence emission ratio (*F*_30 min_/*F*_initial_) of *E. coli* and *B. cereus* treated with NP2 and NPr (125, 250, and 500 μg/mL); *** means *p* <0.001.

Pink et al. reported the importance of electrostatic interactions between protamine and the negatively-charged polysaccharide O-sidechains in bacteria [28]. An increase in electrostatic affinity for the cell surface of targeted bacteria increased the antibacterial efficacy of protamine [29]. The zeta potential of *B. cereus* was −37.03 ± 0.35 mV and for *E. coli* it was −29.30 ± 3.53 mV (Figure 3A). This result suggests that the *B. cereus* had a more negative surface charge than *E. coli*. The surface hydrophobicity of bacteria was tested based on their binding to xylene. The hydrophobic index of *B. cereus* was found to be 0.08 ± 0.03 mV, and the index of *E. coli* was 0.27 ± 0.03 mV. Measurement of hydrophobicity and zeta potential showed that *B. cereus* had more hydrophilic and negatively-charged cellular structure compared to *E. coli*. A more negatively-charged cell should be able to attract a higher amount of positively-charged chitosan nanoparticles (NP) and chitosan/protamine nanoparticles (NPr). Nevertheless, the previously mentioned results showed that *B. cereus* was generally more resistant to the NPr nanoparticles than *E. coli* (Table 3). 

This condition might be caused by different cell wall structures of gram-positive and gram-negative bacteria. The gram-positive cell wall consists of many layers of peptidoglycan whose thickness are generally 30 to 100 nm, whereas the cell wall of gram-negative bacteria is only a few nanometers thick. Gram-negative bacteria have outer membranes at the first layer of the cell wall structures, and the outer membrane is a lipid bilayer composed of glycolipids and lipopolysaccharide. This structure might not provide sufficient protection against protamine. Pink et al. examined the interaction of protamine with gram-negative bacterial membranes [30]. They found that the internalization of protamine by gram-negative bacteria such as *E. coli* was most likely mediated by cation-selective barrel-like proteins, but not the phospholipid bilayer. This may explain the higher susceptibility of *E. coli* to the hybrid nanoparticles (NPrs) compared with *B. cereus*.

### 2.5. Formation of Biofilm-Like Structure

*B. cereus* treated with chitosan/protamine nanoparticles (NPr1, 125 μg/mL protamine) showed biofilm-like structure produced on the surface and bottom of the tube. The control group was observed to develop a bacterial structure at the bottom of the tube. The zeta potential of *B. cereus* was −37.03 ± 0.35 mV and that of *E. coli* was −29.30 ± 3.53 mV (Figure 3A). This result suggested that the *B. cereus* has a more negative surface charge than *E. coli*. Despite having low antimicrobial activity towards gram-positive *B. cereus*, chitosan/protamine nanoparticles (NPr) were found to induce the formation of a biofilm-like structure in this bacteria (Figure 2Ed). NPr nanoparticles also induced a non-motile-like state in *E. coli* (Figure 2Eb), although the result was not as apparent as the effect of chitosan nanoparticles (NP) (Figure 2Ea). These phenomena might be caused by the induction of cyclic diguanylate monophosphate (cyclic di-GMP)-mediated pathways. Previously, it had been reported that cyclic di-GMP was involved in the regulation of motility and biofilm formation [31]. Chitosan nanoparticles might have an impact on these pathways, leading to the induction of non-motile state and production of biofilm.

### 2.6. Production of Extra Polysaccharides

To confirm the above-mentioned inference, six bacteria species (*B. subtilis*, *B. amyloliquefaciens*, *B. pumilus*, *B. megaterium*, *B. cereus*, and *E. coli* K12) were tested on Congo red agar for the production of extra polysaccharides (Figure 3B). Congo red dye was able to stain secreted polysaccharides and showed red color, while brilliant blue dye was able to stain the protein components and appeared blue. Therefore, the red and blue color staining was used as a marker for the visualization of biofilm formation. *B. cereus* was observed to form the largest colonies that were stained with Congo red and brilliant blue dye compared to the other tested bacteria. *E. coli* was shown to develop transparent colonies that were not stained by the dye. The secretion of polysaccharides has been shown to be involved in microbial resistance [32]. It was reported that a polysaccharide matrix provides an effective barrier that restricts the penetration of chemically-reactive biocides and cationic antibiotics [33]. Secreted polysaccharides produced by *B. cereus* might be able to provide some barriers to the nanoparticles and increase the survival rate of the bacterium. In the assay, *B. cereus* was found to produce a higher amount of exopolysaccharide compared to *E. coli* (Figure 3B). The result showed that *B. cereus* developed both red and blue color in its colony. On the other hand, *E. coli* was shown to be transparent and was considered to not produce a biofilm.

Many probiotics in human and animal digestive systems are gram-positive, such as *Lactobacillus* and *Bifidobacterium* genera. In agriculture, many beneficial gram-positive bacteria, such as *Bacillus* and *Streptomyces* have been reported to have roles in decomposition, bio-control of pathogens, bioremediation, and plant growth promotion. The chitosan/protamine nanoparticle (NPr1) was found to have lower inhibition of gram-positive *B. cereus*. It is likely that it also has low inhibition of other beneficial gram-positive bacteria. This nanoparticle should be further investigated to understand its activity and specificity towards pathogenic gram-negative bacteria.

### 2.7. Membrane Permeability

Bacteria were tested with chitosan (125 μg/mL), protamine (125 μg/mL), NP2 (250 μg/mL), NPr1 (250 μg/mL), and control (5 mg/mL NaCl) for membrane permeability. In the *E. coli* assay (Figure 3C), chitosan was observed to increase *A*_260_ and reached a stationary at around 60 minutes. NP2 increased the *A*_260_ higher than chitosan but reached the stationary at around the same time of the chitosan group. NPr1 was observed to increase *A*_260_ and reached stationary at around 110 min. Protamine increased *A*_260_ at the first 20 min, then decreased to negative values towards the end of 120 minutes. In the *B. cereus* assay (Figure 3C), the result showed that chitosan increased the *A*_260_ value rapidly and to a much higher value compared to the other treatment groups in the first 20 min. NP2 and NPr1 were found to increase the *A*_260_ value as high as the control group and decreased slightly until the end of the 120 min. The *A*_260_ was observed to increase slightly in the first 20 min, but then decreased to the initial value at the end of the assay. Protamine was observed to give a fluctuation in *A*_260_ value in the first 80 min, and eventually led to negative values at the end of the assay. The reason why A_260_ is negative is that the Muller-Hinton (MH) Broth medium was used as a blank for initial calibration. The culture medium contains casein and beef extract, which also have absorption at 260 nm. When the bacteria grow, they consume casein and beef extract in the medium, leading to the decrease of the absorbance at 260 nm.

A membrane permeability assay was conducted to study the effect of the different treatments on the integrity of the cell membrane [34]. The increase of *A*_260_ was considered to be the cause of the release of protein or nucleic acids from inside the cell. Treatment groups that showed the increase of *A*_260_ higher than the control group were considered to be able to damage the cell membrane. The results showed that chitosan in polymeric form increased *A*_260_ highly in the first 20 min and maintained this value until the end of the assay. This suggested that chitosan had the activity that led to damage of the bacterial cell membrane. Unlike the chitosan in polymeric form, protamine was observed to have a fluctuation of the *A*_260_ value and reached a negative value at the end of the assay. This result suggests that protamine might not be able to cause the cell membrane damage in *B. cereus*. NP and NPr were shown to increase *A*_260_ slightly higher than the control group. They were not able to break the cell membrane, despite having a concentration two times (250 μg/mL) greater than the chitosan in polymeric form. NPr1 showed a less-steep *A*_260_ value slope change and longer time to reach stationary phase. The results also showed different patterns of *A*_260_ changes from the protamine only group. Taken together, these results suggest that the addition of protamine changed the membrane permeability of the chitosan nanoparticles.

Figure 3D shows the binding affinity of chitosan nanoparticles (NP) and chitosan/protamine nanoparticles (NPr) to *E. coli* and *B. cereus*. There were no differences between the binding (30 min)-to-initial (0 min) fluorescence emission ratio (*F*_30 min_/*F_initial_*) of *E. coli* and *B. cereus* treated with 0.005% fluorescein (the control groups). The group of *E. coli* treated with fluorescein-labeled chitosan nanoparticles (NP) exhibited similar *F*_30 min_/*F*_initial_ to its *B. cereus* counterpart, so the chitosan nanoparticles had the same tendency to bind to *E. coli* and *B. cereus*. However, *F*_30 min_/*F*_initial_ values of the groups of NPr-treated *B. cereus* were lower than those of the nanoparticles-treated *E. coli*, indicating that the NPrs had a higher binding affinity towards *E. coli* than *B. cereus*. These data further explain our previous results showing that MIC and MBC of NPr against *B. cereus* were higher than those against *E. coli*.

Finally, the widespread overuse of antibiotics was reported to cause the presence of a sub-inhibitory concentration in many natural environments, such as sewage water and sludge, rivers, lakes, drinking water, and livestock. This condition is prone to accelerate the emergence and spread of drug-resistant bacteria [35]. Therefore, chitosan/protamine nanoparticles could have the potential as an alternative antibacterial agent to be applied in various fields, such as animal husbandry, plant production, and aquaculture.

## 3. Experimental Section

### 3.1. Materials

Chitosan was purchased from KOYO Chemical Ind., Ltd. (Tokyo, Japan). Protamine, Congo red, Brilliant Blue G, and xylene were purchased from Sigma Chemical Co., Ltd. (St. Louis, MO, USA). Muller-Hinton (MH) Broth and LB media were purchased from Difco Laboratories Inc. (Detroit, MI, USA). Tripolyphosphate (TPP) were purchased from Showa Chemical Industry Co., Ltd. (Tokyo, Japan). *Escherichia coli* (BCRC 51956, K12), *Bacillus amyloliquefaciens* (BCRC 80282, FZB42), and *Bacillus subtilis* (BCRC 10029) were purchased from Bioresource Collection and Research Center (BCRC, Hsinchu, Taiwan). *Bacillus megaterium*, *Bacillus cereus* NCHI37, and *Bacillus pumilus* NCHI14 were our laboratory stock strains.

### 3.2. Preparation of Chitosan-TPP Nanoparticles (NP)

Chitosan stock solution was prepared by adding chitosan powder in acetic acid solution (1% *w*/*v*) and the suspension was stirred overnight to dissolve the powder. The final concentration of the obtained chitosan stock solution was 1% (*w*/*v*). Chitosan nanoparticles were prepared based on an ionic gelation method. Basically, chitosan was mixed with sodium tripolyphosphate (TPP) at a weight ratio of 3:1. The chitosan stock solution was diluted to the required concentration with deionized distilled water (ddH_2_O) and adjusted to pH 4.5. Afterward, an amount of TPP solution was flushed mixed with the chitosan solution to the desired ratio and stirred at 750 rpm for 10 min to obtain the nanoparticles. Three groups of nanoparticles prepared from 250 μg/mL chitosan/ 83.4 μg/mL TPP (NP1), 500 μg/mL chitosan/ 166.7 μg/mL TPP (NP2), and 750 μg/mL chitosan/ 250 μg/mL TPP (NP3) were examined further in this study. 

### 3.3. Preparation of Chitosan/Protamine Nanoparticles (NPrs)

Protamine was used as a cationic antibacterial peptide in this study. The chitosan stock solution was diluted to the required concentration with ddH_2_O. The nanoparticles were prepared by mixing 1 mL of protamine solution with 2 mL chitosan solution and stirred at 750 rpm. Then, an amount of sodium tripolyphosphate was flushed mixed with the chitosan/protamine mixture stirred for 10 min. Three groups of chitosan/protamine NPs prepared from 500 μg/mL chitosan/ 166.7 μg/mL TPP/protamine 125 μg/mL (NPr1), 500 μg/mL chitosan/ 166.7 μg/mL TPP/protamine 250 μg/mL (NPr2), and 500 μg/mL chitosan/ 166.7 μg/mL TPP/protamine 500 μg/mL (NPr3) were examined further in this study. 

### 3.4. Chemical and Physical Properties of Nanoparticles

The nanoparticles’ surface morphologies and sizes in dry state were observed by using a H-7650 transmission electron microscope (TEM, Hitachi, Japan) after applying the nanoparticles to carbon-coated copper grids and drying them. The Fourier transform infrared (FT-IR) spectra were recorded on a Thermo Fisher FTIR spectrophotometer (Waltham, MA, USA) to determine the chemical compositions of the nanoparticles. Measurements of particle size and surface charge were performed using a Zetasizer 3000 (Malvern, UK).

### 3.5. Measurement of Bacterial Zeta Potential

The electrophoretic mobility of the bacterial cells was measured with a Zetasizer 3000 (Malvern, UK) at 25 °C. An aliquot of the freshly harvested bacteria was suspended in 10 mM KCl solution. The concentration of bacterial cells used for zeta potential measurement was adjusted to 1 × 10^5^ cells/mL.

### 3.6. Determination of Minimum Inhibitory Concentration (MIC) and Minimum Bactericidal Concentration (MBC)

The minimum inhibitory concentration (MIC) was determined by a broth dilution method performed in 96-well microtiter plates. Bacterial culture grown to log phase was adjusted to 5 × 10^5^ cells/mL in Muller-Hinton (MH) Broth. Inoculants of 100 μL were mixed with 100 μL of two-fold serial dilutions of different treatment groups and were subsequently incubated at 37 °C for 20 h. The antibacterial activity of NPs and NPrs were determined on the basis of turbidity by a μQuant Scanning Microplate Spectrophotometer (Biotek, Winooski, VT, USA). The measured values were further analyzed in R software (Environment for Statistical Computing (R) 3.2.0, CA, USA) to determine the significant difference between each dilution. The minimum bactericidal concentration (MBC) was determined by spreading 10 μL samples from wells on MH agar plates. The concentration at the highest dilution exhibiting no bacterial growth on agar plates after incubation at 37 °C for 12 h was identified as the MBC. Two independent experiments were performed in triplicate to determine the MIC/MBC values.

### 3.7. Measurement of the Hydrophobicity of Cell Surface

The hydrophobicity of the bacterial cell surface was measured based on the interaction of bacteria with xylene [36]. Bacteria were harvested by centrifugation at 3000 rpm for 20 min, then washed and aliquoted in PBS buffer. The turbidity was adjusted to optical density (OD) 0.4 at 660 nm (*A*_660_ control) using a μQuant Scanning Microplate Spectrophotometer. Approximately 2.5 mL of the bacteria solution was mixed with 1 mL of xylene, the suspension then was vigorously agitated for 2 min, and was allowed to stand for 20 min at room temperature for the separation of two phases. The aqueous phase from the bottom of the tube was removed, then the absorbance was measured (*A*_660_ test). The index of hydrophobicity (HI) was calculated as follows:

HI = (*A*_660_ control − *A*_660_ test) ÷ *A*_660_ control.

### 3.8. Detection of Membrane Integrity

The integrity of bacterial cell membranes was examined by determining the absorbance of the released material (nucleic acids and sugar metabolites) at 260 nm. Overnight bacterial cultures were sub-cultured on fresh LB media and grown for 5 h at 37 °C. The bacteria cells were harvested by centrifugation at 3000 rpm for 20 min, and then washed and re-suspended in 0.5% NaCl solution. The final cell suspension was adjusted to an absorbance of 0.7 at 420 nm (*A*_420_). Approximately 100 μL of bacteria were mixed with 100 μL of different treatment groups (protamine, chitosan, NP, and NPr). Absorbance at 260 nm was monitored with a μQuant Scanning Microplate Spectrophotometer every 5 min for a total time of 120 min. 

### 3.9. Detection of Polysaccharide Formation

The production of curli fimbriae was determined based on the uptake of red color and blue color on NB Congo red plates (NB plates containing 40 μg/mL Congo red and 20 μg/mL Brilliant Blue G). A single colony of tested bacteria was grown overnight in NB medium. Subsequently, the bacteria were transferred to an NB Congo plate with an inoculation loop and cultured at 37 °C overnight.

### 3.10. Detection of Biofilm-Like Structure Formation

Bacteria cell was grown overnight in MH broth medium. The cell concentration was then adjusted to 5 × 10^5^ cells/mL in MH broth medium. One milliliter of bacteria cell then was mixed with 1 mL of 500 μg/mL NPr and incubated at 37 °C for 7 days; the tubes were observed, and the pictures were taken.

### 3.11. In Silico Analysis of Protamine

The amino acid sequence of the major protamine component was taken from the Uniprot database, protamine YI (P69012), YII (P69009), Z (P69011). Calculation of isoelectric point and grand average of hydropathicity (GRAVY) was conducted by Expasy ProtParam. Simulation of the peptide folding was calculated using Pepfold 3.1 peptide structure prediction server. Peptide structure and depiction of the hydrophobicity was drawn using UCSFChimera1.11.

### 3.12. Fluorescence Assay for Nanoparticles Binding to Bacteria

Fluorescein isothiocyanate (FITC)-chitosan conjugate was synthesized by the following process. Briefly, FITC was dissolved completely in DMSO and the FITC solution was subsequently added into chitosan solution at a final concentration of 1 mg/mL. After 12 h of reaction, the FITC-chitosan conjugate was dialyzed to completely remove the residual dyes. The lyophilized fluorescent products were used to prepare chitosan and chitosan/protamine nanoparticles according to the previously mentioned method. The chitosan and chitosan/protamine nanoparticle were incubated with bacterial suspensions for 30 min. The bacterial suspensions were centrifuged at 6000 rpm for 15 min. After centrifugation, the supernatants were collected and the binding (30 min)-to-initial (0 min) fluorescence emission ratio (*F*_30 min_/*F*_initial_) of *E. coli* and *B. cereus* were determined using a Fluodia T70 fluorescence spectrophotometer at excitation (*E*x)/emission (*E*m) = 490/530 nm.

### 3.13. Statistical Analysis

All measurements were replicated three times and data were expressed as the mean ± standard deviation. Statistical analysis was performed with the analysis of variance (ANOVA) procedure using SAS version 9.1 (SAS Institute, Cary, NC, USA). The differences among the experimental data were determined using multiple comparisons of individual means by pairwise *t*-tests using a Bonferroni adjustment.

## 4. Conclusions

In this study, different preparation methods were tested to generate chitosan/protamine nanoparticles. The addition of protamine to chitosan was found to affect the particle size and zeta potential of the hybrid nanoparticles. Protamine was found to increase the antimicrobial activity of chitosan nanoparticles and change the membrane permeability towards *E. coli*. Treatments of chitosan/protamine hybrid nanoparticles were also found to induce the formation of biofilm-like structure in *B. cereus* and non-motile like structure in *E. coli*, which might be correlated to c-di GMP induction which has the potential to inhibit the virulence of pathogens and improve the interaction of plant growth-promoting bacteria. Although the antibacterial activity of the hybrid nanoparticles was lower than silver nanoparticles or commercially produced antibiotics, the nanomaterials developed in this study are not harmful to the environment or human health, and do not cause antibiotic resistance. This property might be further developed to prepare nano-sized antimicrobial agents with a higher activity and specificity towards pathogenic gram-negative bacteria.

## Figures and Tables

**Table 1:** Antimicrobial activities of chitosan polymer against *Escherichia coli* (*E. coli*) and *Bacillus cereus* (*B. cereus*). MBC: minimum bactericidal concentration; MIC: minimum inhibitory concentration.

Chitosan	MIC (µg/mL)			MBC (µg/mL)	
	*E. coli*	*B. cereus*	*E. coli*		*B. cereus*
M1 (80 kDa)	125	62.5–125	250		62.5–125
M2 (200 kDa)	62.5–125	62.5–125	125		62.5–125
M3 (500 kDa)	125	62.5–125	125–250		62.5–125
M4 (1500 kDa)	125–250	62.5–125	125–250		62.5–125

**Table 2:** Size distribution and zeta potential of chitosan (CS) nanoparticles (NPs).

Type of Nanoparticles	Size (nm)	Zeta Potential (mV)
CS NPs		
NP1	78.4 ± 4.01	33.77 ± 1.30
NP2	150.67 ± 3.05	33.63 ± 0.32
NP3	201 ± 3.60	32 ± 1.11
CS/Protamine NPs		
NPr1	114.33 ± 4.16	32.23 ± 0.76
NPr2	84.8 ± 2.07	30.27 ± 0.72
NPr3	79.4 ± 1.90	27.67 ± 1.45

**Table 3:** Antimicrobial activity of chitosan (CS), chitosan nanoparticles (NP), protamine (Pr), and chitosan-protamine nanoparticles (NPr) against *E. coli* and *B. cereus*.

Antimicrobials	MIC (µg/mL)		MBC (µg/mL)	
	*E. coli*	*B. cereus*	*E. coli*	*B. cereus*
Cs only (200 kDa)	62.5–125	62.5–125	125	62.5–125
NP1	31.25–125	125	125	≥125
NP2	31.25–125	125	>250	≥250
NP3	31.25–125	125	125	≥250
Protamine	31.25–62.5	62.5	31.25–62.5	125
NPr1	31.25	>250	31.25–62.5	>250
NPr2	31.25	>250	31.25–62.5	>250
NPr3	31.25	31.25	31.25–62.5	>250
